# Multiple drug hypersensitivity associated with severe cutaneous adverse reaction

**DOI:** 10.1186/2045-7022-4-S3-P135

**Published:** 2014-07-18

**Authors:** Karen Jui Lin Choo, Shiu Ming Pang, Haur Yueh Lee

**Affiliations:** 1 Singapore General Hospital, Dermatology Unit and Allergy Clinic, Singapore; 2 Singapore General Hospital, Dermatology Unit, Singapore

## Background

Multiple Drug Hypersensitivity (MDH) was first described by Sullivan et al in 1989 as drug allergies to two or more chemically different drugs proven by in-vivo or in-vitro testing. Pichler et al added to this description by suggesting that there are two subtypes of MDH: one where sensitisation to different drug classes may occur simultaneously; the other, sequentially - sometimes even years apart.

## Methods

We describe three patients matching the description of MDH.

## Results

We described three patients, aged 47, 68 and 75 with a history of severe cutaneous adverse reaction (SCAR) to non-antibiotic medication (two of the cases were to allopurinol and the other was to omeprazole). All three later developed an exanthem ( 4 to 37 months later) to penicillin-based antibiotics that they were exposed to during the period of 5 to 20 days of their initial SCAR. 1 patient had a positive patch test to the antibiotic.

## Conclusion

Our findings support the hypothesis that concomitant sensitisation to medication(s) given at or around the time of a previous severe cutaneous drug reaction results in multiple drug hypersensitivity syndrome. New or unnecessary medication should be avoided in the initial period following SCAR.

